# Molecular and phylogenetic analyses of a new Amphotropic murine leukemia virus (MuLV-1313)

**DOI:** 10.1186/1743-422X-3-101

**Published:** 2006-12-05

**Authors:** Thomas M Howard, Zhijuan Sheng, Mingwu Wang, Yongchun Wu, Suraiya Rasheed

**Affiliations:** 1 Laboratory of Viral Oncology & Proteomics Research, Department of Pathology Keck School of Medicine University of Southern California Los Angeles, CA 90032-3626, USA; 2 County of Los Angeles Department of Health Services Public Health Programs, HIV-Epidemiology Program 600 S Commonwealth Ave., Suite 805 Los Angeles, CA 90005-4001, USA; 3 Department of Ophthalmology Keck School of Medicine University of Southern California Los Angeles, CA 90032-3626, USA

## Abstract

**Background:**

The amphotropic murine leukemia viruses (MuLV-A's) are naturally occurring, exogenously acquired gammaretroviruses that are indigenous to the Southern California wild mice. These viruses replicate in a wide range of cell types including human cells *in vitro *and they can cause both hematological and neurological disorders in feral as well as in the inbred laboratory mice. Since MuLV-A's also exhibit discrete interference and neutralization properties, the envelope proteins of these viruses have been extremely useful for studying virus-host cell interactions and as vehicles for transfer of foreign genes into a variety of hosts including human cells. However, the genomic structure of any of the several known MuLV-A's has not been established and the evolutionary relationship of amphotropic retroviruses to the numerous exogenous or endogenous MuLV strains remains elusive. Herein we present a complete genetic structure of a novel amphotropic virus designated MuLV-1313 and demonstrate that this retrovirus together with other MuLV-A's belongs to a distinct molecular, biological and phylogenetic class among the MuLV strains isolated from a large number of the laboratory inbred or feral mice.

**Results:**

The host range of MuLV-1313 is similar to the previously isolated MuLV-A's except that this virus replicates efficiently in mammalian as well as in chicken cells. Compared to ENV proteins of other MuLV-A's (4070A, 1504A and 10A-1), the gp70 protein of MuLV-1313 exhibits differences in its signal peptides and the proline-rich hinge regions. However, the MuLV-1313 envelope protein is totally unrelated to those present in a broad range of murine retroviruses that have been isolated from various inbred and feral mice globally. Genetic analysis of the entire MuLV-1313 genome by dot plot analyses, which compares each nucleotide of one genome with the corresponding nucleotide of another, revealed that the genome of this virus, with the exception of the *env *gene, is more closely related to the biologically distinct wild mouse ecotropic retrovirus (Cas-Br-E) isolated from another region of the Southern California, than to any of the 15 MuLV strains whose full-length sequences are present in the GenBank. This finding was corroborated by phylogenetic analyses and hierarchical clustering of the entire genomic sequence of MuLV-1313, which also placed all MULV-A's in a genetically distinct category among the large family of retroviruses isolated from numerous mouse strains globally. Likewise, construction of separate dendrograms for each of the Gag, Pol and Env proteins of MuLV-1313 demonstrated that the amphotropic retroviruses belong to a phylogenetically exclusive group of gammaretroviruses compared to all known MuLV strains.

**Conclusion:**

The molecular, biological and phylogenetic properties of amphotropic retroviruses including MuLV-1313 are distinct compared to a large family of exogenously- or endogenously-transmitted ecotropic, polytropic and xenotropic MuLV strains of the laboratory and feral mice. Further, both the naturally occurring amphotropic and a biologically discrete ecotropic retrovirus of the Southern California wild mice are more closely related to each other on the evolutionary tree than any other mammalian gammaretrovirus indicating a common origin of these viruses. This is the first report of a complete genomic analysis of a unique group of phylogenetically distinct amphotropic virus.

## Background

A large number of genetically transmitted endogenous murine leukemia viruses (MuLVs) and non-genetically acquired exogenous retroviruses have been classified on the basis of their *in vitro *host range, interference and neutralization properties. Regardless of their origin, the gammaretroviruses isolated from a wide variety of inbred or feral mouse strains have been designated as ecotropic (MuLV-E), xenotropic, (MuLV-X), amphotropic (MuLV-A), polytropic, **m**ink **c**ell **f**ocus forming (MCF) and 'modified polytropic' viruses [1-12]. The MuLV-E's are the most common endogenous or exogenously acquired retroviruses of mice and they grow well in mouse or rat cells but not in cells derived from higher primates, humans or other mammals [2]. All MuLV-E strains induce syncytia in a Rous Sarcoma virus transformed, non-producer XC rat cells [13,14]. The xenotropic viruses (MuLV-X) are the genetically transmitted endogenous retroviruses of mice that do not replicate well in mouse cells which produce these viruses, but they grow preferentially in cells of heterologous species, including human and other primate cells [6,7,15]. The polytropic and 'modified polytropic' viruses are endogenous nonecotropic MuLVs that grow in mouse, human and other mammalian cell types [11,12,16]. Most of the polytropic viruses are expressed during leukemogenesis in various inoculated mice and they are called **m**ink **c**ell **f**ocus forming (MCF) as they induce syncytia in the replication defective Kirsten mouse sarcoma virus transformed non-producer, mink cells [17] In contrast, the amphotropic retroviruses do not induce foci in transformed mink cells (i.e. not related to MCF viruses) and they display distinct interference, host range and neutralization patterns from all other endogenous or exogenously acquired, ecotropic, nonecotropic, xenotropic, polytropic or MCF MuLV strains [1,2,4,5,12,18,19].

The proviral DNA sequences of MuLV-A strains are not detected in the DNA from the wild or inbred laboratory mice, indicating that these viruses are not endogenous to the mouse genomes [10,20]. The MuLV-A-related sequences are also not detected in the genomes of numerous avian and mammalian species that normally inhabit various farms from which the MuLV-A harboring wild mice are trapped [20]. Despite numerous studies conducted on wild mice from different regions of the world [12,16,21-23], the amphotropic viruses have been recovered exclusively from the feral mice of Southern California as naturally transmitted infectious agents [1,2,5,10,24].

An interesting phenomenon related to the amphotropic retroviruses (1504A, 4070A and others) is that most of these viruses coexist in nature as mixtures with the biologically distinct ecotropic, XC+ syncytia-inducing retroviruses such as Cas-Br-E (a clone of 1504E), 4070E and others [1,2,10,25]. Further, both types of retroviruses are capable of causing similar diseases in naturally infected feral mice and both can be isolated from the healthy fetal or adult tissues, spontaneous lymphoma/leukemia or from the brain tissues of paralyzed wild mice [2,10,24,26,27]. The amphotropic and ecotropic MuLV components can be separated from the original stocks of virus mixtures by endpoint dilution purification techniques and sequential biological cloning of each virus in human and mouse cells respectively [2,18,28].

The biologically cloned amphotropic and ecotropic retroviruses are quite stable when inoculated in uninfected *wild mice *from areas with low prevalence of exogenously transmitted viruses and both are capable of causing lymphoma as well as paralysis in these animals with no change in their respective host ranges [10]. However, inoculation of inbred mice separately with MuLV-A or MuLV-E strains isolated from wild mice, results in genetic recombination with the endogenous ecotropic, polytropic or xenotropic sequences present in the genomes of the laboratory mice [30,31,29,4]. For example, we have isolated a highly oncogenic amphotropic retrovirus designated 10A-1 by inoculation of a weakly oncogenic amphotropic virus (1504A) isolated from a wild mouse embryo [28]. This virus produces 100% lymphoma in the NIH Swiss mice only in 4–8 weeks and it displays distinct neutralization and interference properties [28]. In addition, at least 50% of NIH Swiss mice inoculated separately with the same 1504-A clone as used for isolating the amphotropic 10A-1 virus, produced ecotropic XC+ MuLV-E [4]. *In vivo *passages of these recovered viruses yielded viruses of ecotropic host range although dual-tropic virus activity was occasionally seen in the spleens but not in the brains or spinal cords of the lymphomatous or paralyzed mice [4,10]. Thus, we have identified three classes of *recombinant *viruses (10A-1-like viruses with amphotropic host range and two genetically distinct recombinants with ecotropic, XC+ host range) that were generated by the recombination of the *env *gene of the exogenously infecting parental virus 1504-A with different endogenous viral or cellular sequences of NIH Swiss mice [4,29,30]. The classic MCF virus was not recovered from any of the laboratory mice inoculated with the wild mouse MuLV-E or MuLV-A [4,10,28].

Our earlier studies had indicated that RNA genomes of all amphotropic viruses including the 10A-I are closely related to each other even though they have recombined with endogenous mouse sequences after inoculation in NIH Swiss mice and they do not induce foci in mink cell (i.e. they are unrelated to MCF viruses in both their *in vitro *and *in vivo *properties) [4,29]. Moreover, RNA genomes of the recovered and cloned MuLV-E isolates from the inbred mice were more divergent from each other than those passaged in the uninfected wild mice [30].

Since the isolation and distribution of the wild mouse retroviruses by our group in early 1970's, the *env *genes and receptors of amphotropic viruses have been studied by many investigators and numerous vectors have been constructed for the delivery of foreign genes in different cell types including human and other primate cells  [30][31-38]. Recent evidence supporting the unusual host range of these viruses has indicated that microdomains in the large cholesterol- rich rafts present in cell membranes are used for early binding events and entry of these viruses in mouse cells [39]. However, a complete genetic structure of any amphotropic MuLV strain is not available and the evolutionary relationships of these retroviruses to a broad range of both endogenous and exogenous MuLV strains have yet to be defined. Herein we present the first complete genetic analysis of a novel amphotropic retrovirus MuLV-1313 and demonstrate that both the amphotropic and the biologically distinct ecotropic MuLV strains isolated from the Southern California feral mice belong to exclusive groups of genetically and phylogenetically related retroviruses and both are distinct from the numerous ecotropic, polytropic and xenotropic MuLV's of the inbred and feral mice from other geographical locations.

## Results and Discussion

### Biological Diversity of MuLV-1313

**Figure 1:**
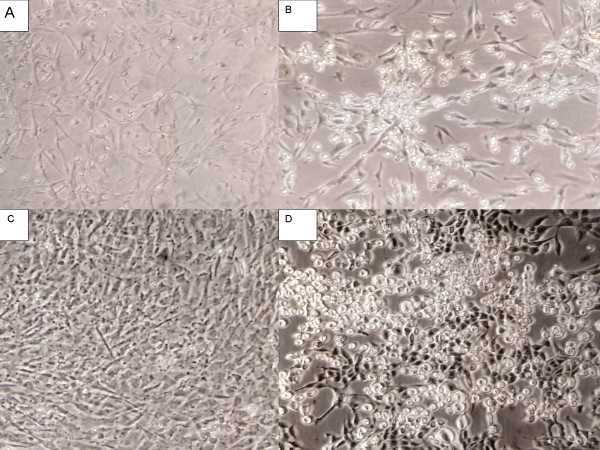
**Chicken and rat embryo cell cultures infected with MuLV-1313 pseudotypes of RSV/*src *genome**. Panel A- Uninfected chicken embryo C/O cells ; Panel B- a culture of same chicken cells as in A, infected with MuLV-1313 pseudotypes of the Rous Sacoma virus (RSV) associated *src *genome ; Panel C- uninfected Fischer rat embryo fibroblasts and Panel D- same rat embryo cells infected with MuLV-1313 pseudotypes of RSV/*src *genome. Note the classical rounded cell morphology of the RSV- transformed cells. The virus infected cultures produced excess helper virus (MuLV-1313) as detected by the presence of reverse transcriptase activity in the culture fluid.

Although the host range of all amphotropic retroviruses is similar, the MuLV-1313 can replicate well in both mammalian and chicken cells (as opposed to the transient transfection of a plasmid-clone) [1,2,4]. This retrovirus is also capable of rescuing the *src *oncogene from the B10-**R**ous **s**arcoma **v**irus (RSV) transformed, non-producer rat cells and the pseudotype virus can transform chicken cells as well as human, bovine, rabbit, dog, raccoon, cat, mink, rat and mouse cells in *vitro *(Figure 1). The infected cells exhibit classical round-cell morphology of RSV-induced transformation and produce excess helper virus as detected by the presence of high levels of reverse transcriptase activity in the culture supernatant. The MulV-1313 (*src*) pseudotype is also able to transform duck embryo cells *in vitro *but the amphotropic virus alone does not replicate as efficiently in duck cells as it does in the chicken cells (S.R., unpublished data).

The choice to characterize MuLV-1313 genomic structure was based on the tenet that this was the only retrovirus that existed in nature as a "pure population" of amphotropic virions since all previously studied MuLV-A's (MuLV1504-A, 4070 and others) were recovered as mixtures with ecotropic retroviruses regardless of their associations with normal embryo tissues, lymphoma, or paralysis [2]. After culturing wild mouse-derived viruses *in vitro*, the amphotropic and ecotropic components had to be separated from the mixtures by biological cloning in different cell types [1,2,4,5]. In contrast, repeat cycles of endpoint dilution cloning of MuLV-1313 strain isolated from lymphoma or other tissues in many different mammalian cell types including human, bovine, dog, rabbit, cat, mink, murine as well as in chicken cells yielded only a single population of amphotropic virions and no ecotropic, xenotropic, polytrophic or MCF-like viral components could be detected in any of these cultures [2].

### Genomic Structure of MuLV-1313 Provirus

The proviral DNA of MuLV-1313 was cloned from the chromosomal DNA of human RD- 1313 cells that were chronically infected with this virus and two fragments that overlapped by 1,934 bps were completely sequenced. The full-length MuLV-1313 genome comprised 8,217 base pairs (bp); a 5,551 bp fragment containing the 5' end of the virus spanned from the U3 region of the 5' LTR to the *pol *gene and a 4,906 bps fragment comprised the 3' end of the genome and extended from the *pol *gene to the U5 region of the 3' LTR. The genomic organization of MuLV-1313 was similar to other replication competent mammalian gammaretroviruses (5'LTR-*gag-pro-pol-env*-3'-LTR) and the proviral DNA spanned from the beginning of the R region of the 5'LTR to the end in the R sequences in the 3' LTR. The base composition of the genome was 26.02% A, 28.55% C, 24.49% G and 20.94% T.

The entire MuLV-1313 genomic sequence was used as a query to align with complete sequences of mammalian gammaretroviruses that were present in various databases. Most significant similarity scores were found to be with the sequences of the MuLV family of retroviruses. The highest score bits (SB) were obtained with the wild mouse ecotropic MuLV Cas-Br-E (SB: 1049) and this was followed by Moloney (SB: 1031), AKV (SB: 920), Rauscher (SB: 912), Friend FB29 (SB: 902), Friend PVC211 (SB: 886), SL3-3 (SB: 880), Friend clone 57 (SB: 870), MCF 1233 (SB: 858), SRS 19-6 (SB: 737). All other MuLV strains including HEMV and MDEV showed low SB scores.

### Regulatory Elements of the MuLV-1313 genome

Alignment of the nucleotide sequences from LTRs of several well-characterized MuLV strains indicated that the total length of the MuLV 1313 LTR was 518 bp with the U3, R and U5 regions comprising 374, 68 and 76 bps respectively. Sequence motifs that were well conserved in the U3 region of the MuLV-1313 LTR included the CAAT box, CGCTT motifs and the TATAA box which comprised the proximal MuLV promoter region. The extreme 5'-end of the U3 region of the MuLV-1313 LTR contained a 13 bp motif (5'-AATGAAAGACCCC-3') that formed one half of the highly conserved inverted repeat element that is also present in the LTR sequences of other mammalian type-C retroviruses [40][41,42]. This sequence motif has been shown to play a key role in the integration of retroviral DNA into the host cellular genome [42]. The U3 region of MuLV 1313 LTR contained 18 bps (5'-AAACAGGATATCTGTGGT-3') that spanned the sequence containing the core region as well as the binding sites for Lvb/Lvt transcription factors. This highly conserved element is also believed to play a key role in the activity of the enhancers of these viruses [40]. Analysis of the U3 region of MuLV-1313 LTR's indicated that it has several additional control regions. These included the upstream sequences designated as the negative control region and a single copy of the 75 bp MuLV enhancer sequence which contained regulatory elements such as the glucocorticoid response element (GRE), core element, GC-rich region and binding sites for different transcription factors (Lvb/Lvt and NF-1).

The R region of the MuLV-1313 LTR contained two important elements. The first was a 28-nucleotide motif (5'-GCGCCAGTCCTCCGATAGACTGAGTCGC-3') located at the extreme 5' end of the R region, which is highly conserved among the mammalian gammaretroviruses. This motif is predicted to form a stable stem-loop structure that would be present at the 5' ends of all RNA transcripts generated from the LTR [41]. This element has also been shown to be necessary for maximal activity of the MuLV- SL3 LTR by influencing the initiation rate of viral transcripts from the MuLV promoter [41]. Immediately downstream from this element was a consensus poly-adenylation signal sequence (AATAAA). The second important element of the MuLV-1313 LTR was a 13 bp inverted repeat (5'-GGGGTCTTTCATT-3') located at the extreme 3' end of the U5 region. Most retroviruses use this element for the integration of proviral DNA's in the host chromosomal DNA. Immediately downstream of the U5 region (5'-TGGGGGCTCGTCCGGGAT-3') was a sequence complementary to the 3' end of tRNA^Pro ^that was identified as a minus strand primer binding site for the first strand DNA synthesis.

### Structural Analysis of the MuLV-1313 Gag/Pro/Pol Polyprotein

The Gag/Pro/Pol precursor protein (Pr180) of MuLV-1313 consisted of 1736 amino acid (aa) residues that were encoded by 5211 bp (from nt 620 to 5830). The polyprotein could be subdivided into the matrix (MA or p15), pp12, capsid (CA or p30) and nucleocapsid (NC or p10). The *pol *gene of MuLV-1313 encoded protease (Pro) and Pol (reverse transcriptase and integrase proteins), which were located in the same continuous open reading frame as the *gag *gene. A stretch of nucleotides located immediately downstream of the stop codon for the Gag shared sequence similarity with a motif found in an analogous position in the Moloney MuLV genome. This region is believed to form a stem-loop structure in the RNA form of the genome and it plays a major role in the suppression of the *gag *termination codon during translation in order to produce the Pr180 Gag-Pro-Pol polyprotein [43]. In MuLV-1313 a precursor Gag/Pro/Pol fusion protein may be expressed via suppression of the *gag *amber termination codon present between nucleotides 2227 and 2230 by a glutamine-charged tRNA [44].

Although the MuLV-1313 Gag-Pro-Pol polyprotein displays similarities of the deduced amino acid residues located at the potential protease cleavage sites to those found in Moloney MuLV [45], several differences were noted: (*i*) an alanine instead of a threonine residue was located immediately after the NC/PR cleavage site, (*ii*) an asparagine instead of a glutamine residue was present 3 residues after the PR/RT cleavage site, (*iii*) a threonine in place of a serine residue could be identified 5 residues after the RT/IN cleavage site, (*iv*) the fourth amino acid residue after the p12/CA protease cleavage site in MuLV-1313 Gag protein could be predicted to be a serine residue instead of an alanine as observed in Moloney MuLV.

The deduced NC (p10) protein of MuLV 1313 contains one canonical retroviral 'CCHC' or Cys-His motif (C-X_2_-C-X_4_-H-X_4_-C) that is considered to be essential for the encapsidation of the viral RNA genome during the virus assembly. The 'HHCC' domain (H-X_(3–7)_-H-X_(23–32)_-C-X_2_-C) that has been shown to be important for retroviral integration in the host chromosomal DNA could be located at the amino terminus of the MuLV-1313 integrase protein in the form of H-X_3_-H-X_32_-C-X_2_-C. A second motif, designated 'DDE' (D-X_(39–58)_-D-X_35_-E), was also present in the MuLV-1313 integrase protein in the form of D-X_39_-D-X_35_-E. This motif has been localized in the catalytic core domain of these proteins and it is universally conserved among integrases of retroviruses as well as retrotransposons.

The p15 matrix (MA) domain of MuLV-1313 exhibited a glycine residue located in the second amino acid position (G2), which is important for viral capsid assembly of many viruses particularly when the Gag proteins are targeted to the cell membrane through myristylation of this residue [46]. We have also located sequences encoding two consecutive amino acid residues Gln and Arg at positions 109 and 110 of the MuLV-1313 CA (p30) protein that are present primarily in N-tropic MuLV strains (i.e., viruses that replicate efficiently in cells derived from NIH Swiss mice). In contrast, the B-tropic MuLV strains (i.e., those that grow preferentially in cells derived from the BALB/c mice) contain Thr-Glu residues in the corresponding positions [3,47,48].Thus the genetic structure of the MuLV-1313 genome is discrete compared to the 27 MuLV genomes that have either been partially or completely sequenced.

### Genetic Structure of the MuLV-1313 ENV Protein

The *env *gene of MuLV-1313 was located in the -1 frame with respect to the Gag and Pol proteins of this virus and it comprised 1965 bp. Several structural domains were identified in the MuLV-1313 Env precursor protein of 654 aa residues (designated Pr80) ; (*i*) an approximately 30 aa amino terminal signal peptide; (*ii*) a 200 aa receptor binding domain containing two hypervariable regions, designated VRA and VRB [49,50]; (*iii*) a proline-rich region ranging between 45 to 59 aa in length and composed of a highly conserved N-terminal sequence and a hypervariable C-terminal sequence; (*iv*) about 160 aa carboxy terminal portion of the surface (SU) protein; (*v*) a transmembrane (TM) ectodomain harboring a sequence at its amino terminus and a heptad repeat domain that may be involved in the fusion of the viral envelope to the cell membrane; (*vi*) a membrane spanning domain and (*vii*) a C-terminal p2E or R-peptide cytoplasmic tail.

**Figure 2:**
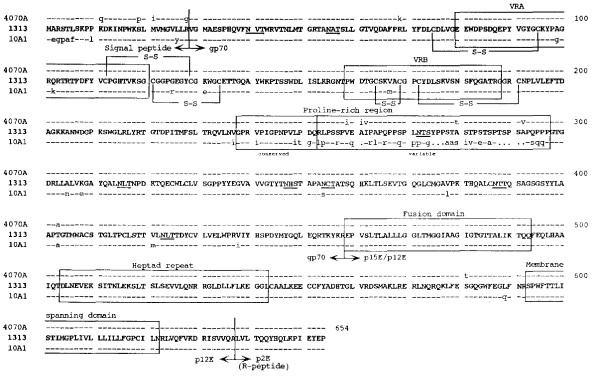
**Relationship of MuLV 1313 gPr80 Env protein with those of other amphotropic MuLV's**. All deduced amino acid sequences of the MuLV 1313 gPr80 Env proteins and related amphotropic MuLV strains 4070A, and 10A-I were aligned using progressive, pair-wise alignments implemented in the Pileup and the Gap programs of the Wisconsin Package (version 9.0), Genetics Computer Group (GCG), Madison, WI [63-67, 80]. The amino acid sequences of MuLV 1313 Env protein are shown in its entirety (represented in bold, capital letters using the standard single-letter symbols). Amino acid positions that are in total agreement with MuLV 1313 Env are indicated with a dash (-) and differences are represented as small letters. Periods (·) in the 10A-I Env protein sequence indicate spaces that were introduced to maximize the alignment. Boundaries of the Env signal peptide (Leader) and the mature processed proteins are labeled and indicated with bold vertical lines. Major landmarks of the extracellular gp70 surface (SU) protein include the (*i*) variable region A (VRA), (*ii*) VRB and (*iii*) proline-rich region. Major landmarks of the p15E transmembrane™ protein include the (*i*) fusion, (*ii*) heptad repeat, (*iii*) membrane spanning and (*iv*) the R-peptide or p2E domains. Eight potential N-linked glycosylation sites (N, X, S/T) in the SU proteins of the MuLV 1313 and 4070A are underlined. Disulfide linkages shown at the N-terminus of SU are based on those deduced for the polytropic envelope protein [81]. The boundaries of the various elements included in this diagram are based on previously published work [51, 82, 83]. Accession numbers for each of the *env *genes used in this analysis are MuLV 1313 (AF411814), M33469 for the naturally occurring amphotropic virus 4070 and M33470 for the recombinant amphotropic virus 10A-1.

The host range, neutralization and interference patterns of various MuLV subgroups have been associated with differences in the viral envelope glycoproteins and their interactions with specific cellular receptor(s) used for viral entry  [4,30-32][33-37,51-56][57]. Since the sequence of the entire genome of an amphotropic virus was not available for comparison with the sequences of the MuLV-1313 genome, this evaluation was restricted to the amphotropic *env *gene only. A search of the GenBank database by the use of the BLAST-P algorithm indicated that the deduced amino acid sequence of MuLV-1313 *env *ORF shared the highest degree of sequence similarity (96.8%) to the amphotropic 4070A (Score bits (SB): 1177) and the recombinant amphotropic virus 10A-1 (94.3%) with two gaps inserted (SB: 1118). However, compared to 4070A Env protein, 12 aa residues were different between the two proteins (Figure-2). Eleven of the twelve aa differences were found in the SU (gp70) protein and one aa difference was found in the TM protein (p15E). Four of these differences were observed in the signal peptide region (Q11K, P19S, I21M and G28R), five in the hypervariable C-terminal sequence of the proline rich region (PRO) (I259V, I261A, V262I, T280A and V292A) and two aa differences could be located between the signal peptide (K69R) and between the proline-rich region and its C-terminus (A403T). No aa residue differences were found in either VRA or VRB regions of their receptor binding domains or in the (eight) potential N-glycosylation sites (N-X-S/T) or positions (all located in the SU protein) of 4070A and MuLV-1313 Env proteins. The only aa residue difference between the TM proteins of 4070A and 1313 was located between the heptad repeat and the membrane spanning domains (T581S) (Figure 2). These domains have been reported to be involved in inducing conformational changes and recognition of cellular receptors for both naturally occurring 4070A and the recombinant amphotropic virus 10A-1 (Figure -2) [53-55][56-60].

An interaction between MuLV particles and specific cell surface receptors appear to depend primarily on two variable regions, designated VRA and VRB, that are located in the amino-terminal domain of the MuLV SU proteins [49,50]. However, a comparison of MuLV-1313 SU protein with those of other amphotropic MuLVs indicates that the growth of this virus in both avian and mammalian cells can not be attributed to these receptor-binding domains since no amino acid differences are noted in VRA and VRB regions of MuLV-1313 and those of 4070A which does not grow in chicken cells. Recently, substantial evidence has been presented for the caveola-dependent endocytosis and binding of MuLV-A to mouse cells by the use of microdomains of their lipid rafts [38,39]. To study this phenomenon we have constructed vectors that express high levels of MuLV-1313 Env proteins *in vitro *(Howard and Rasheed unpublished data) since it is possible that other determinants besides VRA and VRB regions of the MuLV-1313 SU proteins may be involved in the binding of MuLV-1313 viral envelope to cellular membranes and replication in different cell types including avian cells.

The MuLV 1313 Env protein was also different from the highly oncogenic recombinant amphotropic MuLV 10A-1. Comparison of the complete Env sequences of the two viruses indicated that forty-six residues were different between the two proteins and all aa differences with the exception of one were located in their gp70 (SU) domains (Figure-2). These differences were not randomly distributed but were predominantly localized in two areas of this protein, namely the signal peptide region (7 aa residue differences) and the proline-rich region, especially in the variable subdomains. In addition, 2 residues in the VRA region and 1 aa difference in VRB domain were found to be different between MuLV-1313 and 10A-1 Env proteins (Figure 2).

Compared to the polytropic or MCF-type and xenotropic interference groups of viruses, the MuLV-1313 *env *gene showed significant number of gaps (six to seven) and overall similarities of 73.5% to 75.0%. Of all the MuLVs isolated from various mouse strains globally, the 1313 *env *gene shared the least similarity with the ecotropic MuLVs isolated from the inbred or feral mice including those from the Southern California (Cas-Br-E-MuLV) (54% to 65.9% with 10 to 14 gaps).

### Comparison of Full-length MuLV Genomes

Prior to this study a complete genomic structure or the RT or the Gag sequences of any amphotropic virus was not known and only *env *gene had been studied. We have therefore analyzed the whole genomic sequence of MuLV-1313 and in addition evaluated each of its genes separately in comparison with those present in all known MuLV strains whose complete or partial sequences are available in the global databases. Currently 15 full-length MuLV sequences including those of the MuLV-1313 genome are available in the GenBank. All 15 viral genomes were analyzed by two separate methods: 1) DOT Matrix analyses in which each of the 8,217 bps of MuLV-1313 were compared with the corresponding nucleotide of another MuLV strains and 2) phylogenetic analysis by construction of dendrogram using full-length sequences of all 15 MuLV's.

**Figure 3:**
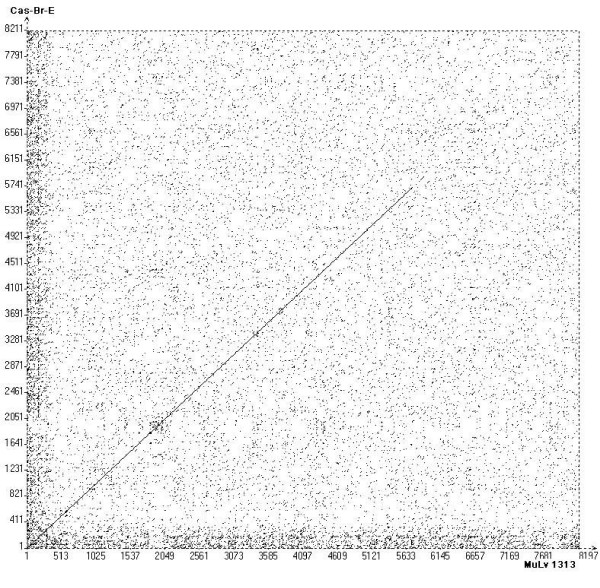
**Dot Martrix analyses of the whole MuLV-1313 genome**. Dot plots of the MuLV-1313 genome (GenBank accession number AF411814) were constructed utilizing the COMPARE which produced files of 15621 points of full-length MuLV genomes. Dot matrix was constructed using DOTPLOT programs of the Wisconsin Package, Version 9.0, Genetics Computer Group (GCG), Madison WI and Vector NTI (Invitrogen, Carlsbad, California) tool with windows setting at 21 and stringency at 44. This analysis compares each nucleotide position with the corresponding position of another genome (Dot). Solid diagonal line represents similarity and broken lines indicate gaps. Although Dot-Matrix analyses were performed on several MuLV strains, viruses that showed high similarity scores are shown in **panels A, B and C (see Additional File 2). Dot Matrix **analyses of full-length genomic sequences shown include ; **Panel A, **Cas-Br-E [25] (X57540); **Panel B, **AKV (J01998), and **Panel C, **Moloney (J02255). The highest nucleotide similarity is observed with the CAS-Br-E ecotropic virus isolated from a Southern California Wild mouse with paralysis **(Panel A). **This is followed by Moloney [8] and AKV MuLV strains [85] **(Panels B & C respectively)**. Note, the *env *sequences of MuLV-1313 are totally unrelated to all three viruses shown by large gap in this area of the diagonal line. In addition, note the numerous broken lines in *gag *and *pol *regions of the Moloney and AKV MuLV genomes.

The Dot Matrix analyses were performed to clearly establish relationship of the entire MuLV-1313 genome to the known MuLV strains that have been completely sequenced. This comprehensive analysis indicated that the MuLV-1313 genome shared the highest nucleotide sequence similarities throughout the Gag and Pol proteins (*not *the ENV) of the biologically distinct ecotropic retrovirus Cas-Br-E (a clonal derivative of MuLV 1504E) isolated from a paralytic wild mouse from Southern California locale (Figure-3 panel A). Although there is no sequence correspondence between the *env *genes of MuLV-1313 and Cas-Br-E, the similarity of the *gag *and *pol *genes was totally unexpected.

Since the host-range, interference and neutralization properties of all exogenous and endogenous ecotropic viruses present in the inbred or the wild mice are similar and totally distinct from MuLV-1313 or other amphotropic viruses, the genetic make up of the wild mouse ecotropic MuLV Cas-Br-E would be expected to be similar to other known ecotropic viruses such as Moloney, AKR and other MuLV strains [2,11,12,18,61,62]. However, as can be seen from Figure 3 Panels B & C (See additional file 2), all these ecotropic and nonecotropic MuLV strains show frequent breaks and shifts in the diagonal dot plots indicating only a distant relationship of their *gag *and *pol *genescompared to those of amphotropic and ecotropic (Cas-Br-E) retroviruses from California wild mice.

**Figure 4:**
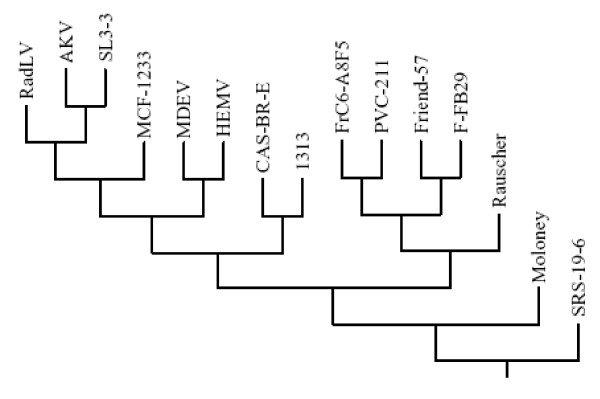
**Phylogenetic analyses of full Length MuLV genomes**. Full-length nucleotide sequences as well as deduced amino acid sequences of 15 MuLV strains including MuLV-1313 were aligned and all gaps were stripped from the alignments before the phylogenetic trees were constructed and bootstrapping was set at 1000. Phylogenetic analyses were performed using PHYLIP [66, 71]. PHYLIP packages SEQBOOT, PROTDIST, DNADIST, NEIGHBOR, CONSENSE, and DRAWGRAM. The original data set was first analyzed by SEQBOOT which produced 100 bootstrapped data sets. The distance matrices on these data sets were achieved using PROTDIST for amino acid sequences and DNADIST for nucleotide sequences. The distance matrices were joined using NEIGHBOR. The tree files from NEIGHBOR were then applied with CONSENSE and the consensus tree was drawn using DRAWGRAM. Multiple sequence alignment were made using Vector NTI (Invitrogen, Carlsbad, California) with default gap opening penalty of 15 and default gap extension penalty of 6.66. Full length genomes used in the construction of the dendrogram included; AKV MuLV (J01998), MuLV 1313 (AF411814), Cas-Br-E MuLV (X57540), Friend-57 MuLV (X02794), Friend FB29 MuLV (Z11128), Friend PVC211 MuLV (M93134), Friend (FrC6-A8F5 D88386), mink cell focus-forming virus 1233 (MCF1233, U13766), Moloney MuLV (J02255), radiation leukemia virus (RadLV, K03363), Rauscher MuLV (Rauscher, U94692), SL3-3 MuLV (AF169256), solid-type reticulum cell sarcoma 19-6 MuLV (SRS 19-6, AF019230), HEMV (AY818896) and MDEV (AF053745). Note that both the amphotropic MuLV-1313 and ecotropic Cas-Br-E MuLV of the Southern California feral mice arise from a separate node of the phylogenetic tree indicating their evolutionary relationship.

Alignment of coding sequences from 15 available full-length MuLV genomes and maximum-likelihood phylogenetic analysis confirmed the findings of the dot matrix plots and showed that both Cas-Br-E and MuLV-1313 arose from a single node and then separated on two branches of the tree due to differences in their ENV (see next section and Figure-4). The other 13 MuLV strains were positioned on either sides of these two branches and clearly segregated the naturally occurring retroviruses of the Southern California wild mice from those isolated from the inbred or other feral mice globally.

### Phylogenetic Analyses of MuLV-1313 GAG and POL Proteins

**Figure 5:**
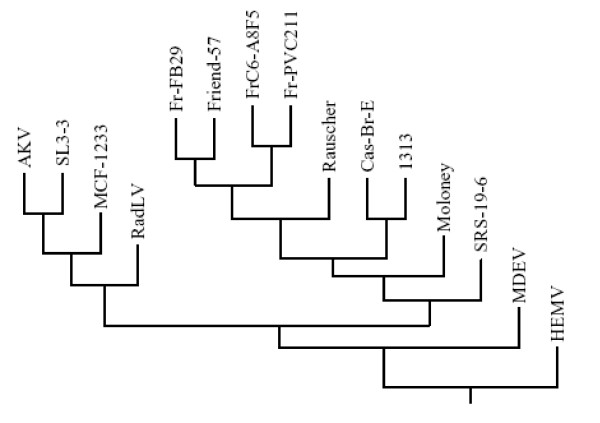
**The phylogenetic tree of MuLV *gag *genes**. Dendrogram was constructed using deduced amino acid sequences of the entire *gag *gene of MuLV-1313 and 14 other full-length Gag sequences available in the databases. Note the evolutionary relationship of the highly conserved *gag *genes of the ecotropic Cas-Br-E and amphotropic MuLV-1313 viruses isolated from the wild mice of the Southern California regardless of their differences in their host range. Sequences used in the analysis include: AKV MuLV (J01998), MuLV- 1313 (AF411814), Cas-Br-E MuLV (X57540), Friend-57 MuLV (X02794), Friend FB29 MuLV (Z11128), Friend PVC211 MuLV (M93134), Friend (FrC6-A8F5 D88386), mink cell focus-forming virus 1233 (MCF1233, U13766), Moloney MuLV (J02255), radiation leukemia virus (RadLV, K03363), Rauscher MuLV (Rauscher, U94692), SL3-3 MuLV (AF169256) and solid-type reticulum cell sarcoma 19-6 MuLV (SRS 19-6, AF019230), HEMV (AY818896) and MDEV (AF053745). Note the segregation of MuLV- 1313 and Cas-Br-E MuLV on a distinct node of the phylogenetic tree.

In general, evolutionary analysis of retroviruses is conducted by the use of amino acid sequences of the highly conserved reverse transcriptase (RT) regions of their *pol *genes. To more specifically define the evolutionary relationship of MuLV-1313 to other MuLV strains, we analyzed each encoded protein of all three MuLV-1313 genes (*gag*, *pol *and *env*) separately. The MuLV-1313 protein sequence was aligned with the corresponding sequences derived from a large family of both exogenous and endogenous retroviruses that have been isolated from different mouse strains and hierarchical clustering dendrograms were constructed separately for each gene  [63][64-67]. Analyses of different regions of the Gag protein (i.e. P15/MA or P30/CA) or the entire sequence of the MuLV-1313 *gag *gene segregated MuLV-1313 together with Cas-Br-E into distinct branches of a common phylogenetic node indicating that these two wild mouse retroviruses are genetically related (Figure 5).

**Figure 6:**
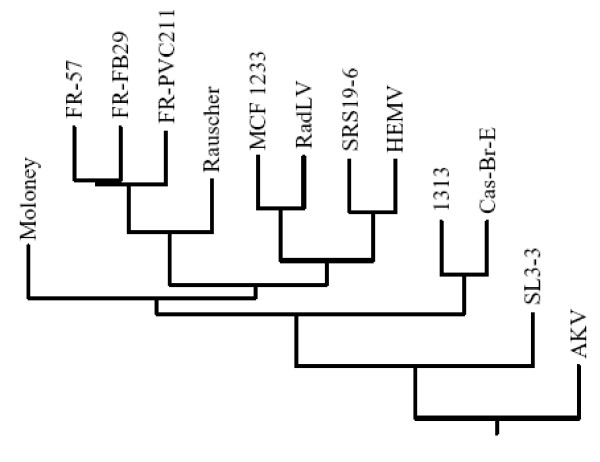
**Evolutionary relationship between highly conserved MuLV *pol *genes. **Dendrogram was constructed from a 576 bp region of the MuLV *pol *gene encoding the highly conserved domains 1 through 7 of the retroviral reverse transcriptase protein [67] by using the neighbor-joining method. No gaps were introduced in any of the respective sequences to maintain the final alignment. Horizontal branch lengths are drawn to scale. Sequences from 13 highly conserved regions of 13 viruses used in the analysis include: MuLV 1313 (AF411814), Cas-Br-E MuLV (X57540), AKV MuLV (J01998), Friend-57 MuLV (X02794), Friend FB29 MuLV (Z11128), Friend PVC211 MuLV (M93134), mink cell focus-forming virus 1233 (MCF1233, U13766), Moloney MuLV (J02255), radiation leukemia virus (RadLV, K03363), Rauscher MuLV (Rauscher, U94692), SL3-3 MuLV (AF169256) and solid-type reticulum cell sarcoma 19-6 MuLV (SRS 19-6, AF019230) and HEMV (AY818896). Comparison of highly conserved *pol *sequences also indicated that MuLV 1313 is distinct from all other MuLV strains and is related to Cas-Br-E MuLV.

To construct the *pol *tree for MuLV-1313 we have selected a region of this gene that encompasses the highly conserved domains 1–7 of the reverse transcriptase (RT) protein encoded by a 534 bp sequence [67]. In previous studies this region had been used for phylogenetic analyses of all retroviruses [67]. The 5' half of the RT was the only region of the MuLV-1313 genome that shared significant sequence similarity (>90%) to this gene from the 12 MuLV strains. This analysis also indicated that the MuLV-1313 *pol *gene was more closely related to the Cas-Br-E strain of MuLV the ecotropic virus from the wild mice of Southern California than to other MuLV strain as both of these viruses segregated from a single node (Figure 6). Additionally, the separation of MuLV-1313 and Cas-Br-E from the other MuLV strains appears to have taken place early in the evolution of these retroviruses (Figure-6).

### Genetic Relationship of MuLV 1313 ENV to those of other mammalian Gammaretroviruses

**Figure 7:**
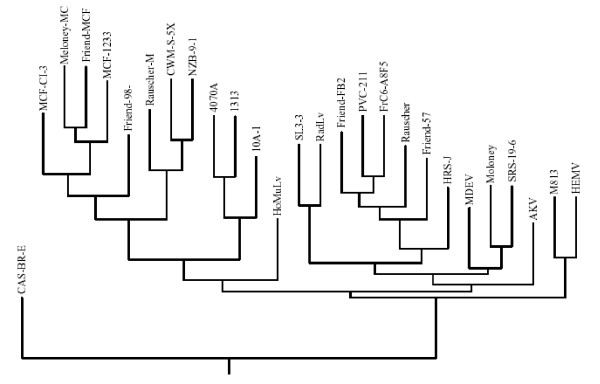
**Phylogenetic analyses of amino acid sequences of complete MuLV *env *genes**. The *env *genes of 26 MuLV strains were compared with those of MuLV 1313(AF411814) and the evolutionary tree was constructed using deduced amino acid sequences of full-length MuLV *env *genes and gaps were stripped from the alignment prior to construction of the final tree. Viruses included are: Friend FrC6-A8F5 MuLV (D88386); Friend PVC-211 MuLV (M93134); Rauscher MuLV (U94692); Friend SFFV-spleen focus-forming virus (AF030173); Friend MuLV (X02794); SRS 19-6 MuLV (AF019230) [82]; Moloney MuLV (J02255) [85]; AKV MuLV(J01998) ; Cas-Br-E MuLV (X57540) ; HoMuLV-*Mus hortalanus *murine leukemia virus (M26527) ; 4070A MuLV (M33469) ; 1313 MuLV(AF411814); 10A1 MuLV (M33470); NZB-9-1 MuLV (K02730); CWM-S-5X MuLV (M59793); HRS/J MuLV (M17326); Friend 98 MCF-mink cell focus-forming virus (AF133256); Friend MCF (X01679); Rauscher MCF (M10100); Moloney MCF (J02254); CI-3 MCF (K02725), 1233 MCF (U13766), MDEV (AF053745), HEMV (AY818896), M813 (AF327437) RadLV (K03363) and SL3-3 (AF16925). Note the unique separation of amphotropic viruses on a single node with a distinct branch for MuLV-1313.

Although our studies and those of others had previously shown the distinctive nature of the amphotropic *env *gene [2,31,32,68][69], the present analysis compares all available ENV protein sequences of the amphotropic, ecotropic, "dual-tropic", polytropic, MCF, "modified polytropic", xenotropic and 10A-1 recombinant amphotropic viruses (Figure-7). Phylogenetic analysis and comparisons of amino acid sequences of the MuLV-1313 ENV protein with 26 other MuLV strains representing each of the major *in vitro *host range, neutralization and interference groups of MuLV strains indicated that the MuLV 1313 Env protein is most closely related to that of the amphotropic MuLV 4070A and these two viruses together with the related 10A-I recombinant amphotropic strain formed a unique node on the MuLV Env evolutionary tree suggesting the uniqueness of their *env *genes among all other members of the MuLV family (Figure-7). Further, the Env analyses clearly positioned the amphotropic retroviruses between the two distinct groups, the nonecotropic, polytropic and MCF-type of viruses on one side and all ecotropic viruses except Cas-Br-E (the only ecotropic virus sequenced from the Southern California wild mouse on the other (Figure-7).

**Figure 8:**
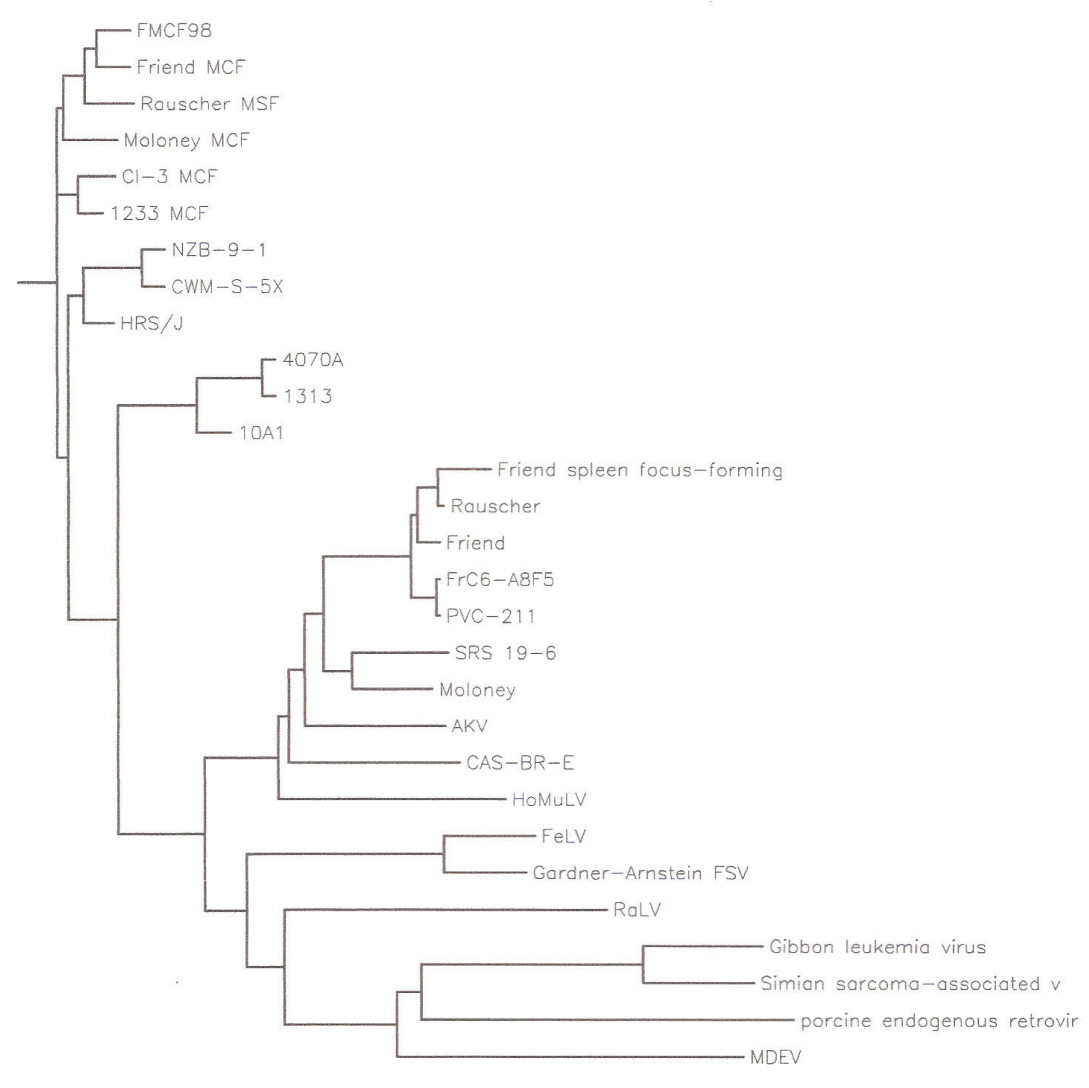
**Phylogenetic relationship of MuLV 1313 to various mammalian gammaretroviruses. **This analysis utilized full-length or nearly full-length Env proteins 29 retroviruses isolated from mouse, rat, cat, pig, monkey and gibbon ape. The original multiple sequence alignment was created with Clustal W and the rooted tree phenogram was constructed using pre-aligned sequences and PHYLIP [64-66, 71, 80]. Abbreviations and accession numbers (in brackets) of viruses used in the phenogram are according to the GenBank database SSAV-simian sarcoma-associated virus (AF055064); GALV-gibbon ape leukemia virus (M26927); PERV-porcine endogenous virus (Y17013); MDEV-*Mus dunni *endogenous virus (AF053745); RaLV-rat leukemia virus (M77194), FeLV A-feline leukemia virus, subgroup A (M18247); FeLV B-feline leukemia virus, subgroup B (Gardner-Arnstein strain) (X00188); MuLV-murine leukemia virus MuLV 1313 (AF411814); Friend FrC6-A8F5 MuLV (D88386); Friend PVC-211 MuLV (M93134); Rauscher MuLV (U94692); Friend SFFV-spleen focus-forming virus (AF030173); Friend MuLV (X02794); SRS 19-6 MuLV (AF019230) [83]; Moloney MuLV (J02255); AKV MuLV(J01998) ; Cas-Br-E MuLV (X57540) ; HoMuLV-*Mus hortalanus *murine leukemia virus (M26527) ; 4070A MuLV (M33469) ; 10A1 MuLV (M33470); NZB-9-1 MuLV (K02730); CWM-S-5X MuLV (M59793); HRS/J MuLV (M17326); Friend 98 MCF-mink cell focus-forming virus (AF133256); Friend MCF (X01679); Moloney MCF (J02254); CI-3 MCF (K02725), 1233 MCF (U13766), MDEV (AF053745), RadLV (K03363) and SL3-3 (AF16925)

The origin of amphotropic MuLV's is not known and sequences related to these viruses are not present in the germ-line of inbred or feral mice indicating that MuLV-A's are not genetically transmitted retroviruses and they spread as infectious agents among the Southern California wild mice [10,20,69]. Analysis of cellular DNA's of many mammalian and avian species also failed to identify MuLV-A-related sequences in their genomes arguing against an inter-species transmission [20]. To establish a genetic relationship of MuLV-1313 genome with other mammalian gammaretroviruses, we constructed phenogram-like rooted tree that compares maximum likelihood relationships to other retroviruses regardless of species of origin (such as cats and primates) and includes many genetic, taxonomic and other characteristics to define overall association between mammalian retroviruses [66,70,71]. This analysis clearly placed the amphotropic retroviruses in a unique position among numerous mammalian retroviruses including those from the rat, cat and primates (Figure-8). These results indicate that amphotropic viruses segregated from the rest of MuLV strains a long time ago in the evolutionary scale and remained as infectious agents among the wild mouse population of the Southern California.

### The MuLV-1313 is Not a Recombinant Retrovirus

Although the phylogenetic analyses of each of the three retroviral genes separately has revealed that regardless of the genetic sequences used (*gag, pol or Env*) for the construction of each dendrogram, both the amphotropic and ecotropic viruses from the feral mice of Southern California segregate from a unique node into their respective clades or branches of the evolutionary tree (Figures 5, 6, 7&8). However, most of the retroviral genomes are genetically diverse due to a high error rate of the polymerase activity during reverse transcription and/or exchange of genetic material co-packaged within the same virion leading to the recombination between two slightly variable viral genomes [72][73-75]. To test if MuLV-1313 genome is chimeric due to any genetic recombination between different but related viral genomes, we have used a program called SimPlot that was originally designed to detect evidence of intersubtype recombination among various human immunodeficiency virus (HIV) genomes [73]. This program has been extremely useful in classifying chimeric genomes of various HIV-1 and HIV-2 subtypes and clades isolated globally [72][73-75].

**Figure 9:**
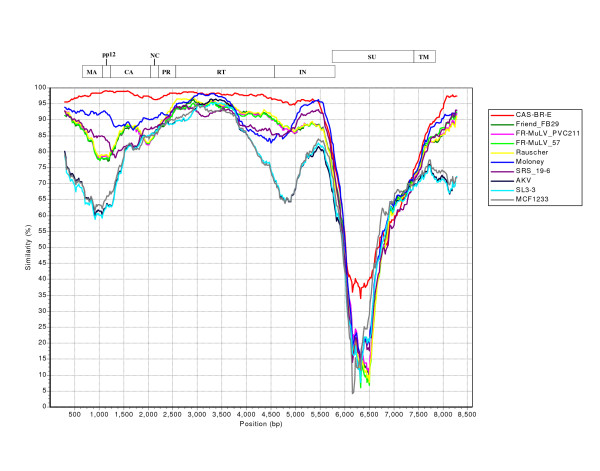
**Similarity Plot of Full-length MuLV genomes: **Plots of similarity between the MuLV 1313 genome (the 'query' sequence) and 10 representative, full-length MuLV genomes (the 'reference' sequences) present in the GenBank database were generated by SimPlot [72]. This program plots similarity versus position and calculates percent identity of a query sequence to a panel of reference sequences after their alignment in a sliding window, which is moved across the alignment in steps. The window and step sizes are adjustable. Prior to this analysis, an alignment of the full-length MuLV genomes was generated by the Clustal W program [64, 80]. Each curve is a comparison between the query genome being analyzed and one of the reference genomes after an alignment of the genomes has been made and positions containing gaps within the alignment were removed. Sequences used in the analysis include: AKV MuLV (AKV, J01998), Cas-Br-E MuLV (CAS-BR-E, X57540), Friend-57 MuLV (FR-MuLV 57, X02794), Friend FB29 MuLV (FR-MuLV FB29, Z11128), Friend PVC211 MuLV (FR-MuLV PVC211, M93134), mink cell focus-forming virus 1233 (MCF1233, U13766), Moloney MuLV (Moloney, J02255), Rauscher MuLV (Rauscher, U94692), SL3-3 MuLV (SL3-3, AF169256) and solid-type reticulum cell sarcoma 19-6 MuLV (SRS 19-6, AF019230). All 'reference' sequences used in the analysis with the exception of the polytropic MCF1233 display ecotropic *in-vitro *host range. Above the curves is a schematic diagram (drawn to scale) showing the relative positions of the coding regions of the *gag *(MA, pp12, CA and CA), *pol *(PR, RT and IN) and *env *(SU and TM) genes of the MuLV genome.

Since recombination in various regions of a viral genome can be recognized only by the genetic analyses of full-length sequences we have used the SimPlot program to identify potential breakpoints for *any *recombination in the entire MuLV-1313 genome in comparison with other full-length MuLV sequences that are available in the database. This analysis did not reveal any breakpoints in the MuLV-1313 genome compared to complete sequences of 14 other MuLV strains indicating that the MuLV-1313 genome is not a recombinant of different genetic sequences derived from other MuLV genomes (Figure-9 and see additional file 1). Without a doubt, this analysis demonstrated that the *RT *and *gag *genes of both MuLV-1313 and the biologically distinct ecotropic virus (Cas-Br-E) from the Southern California wild mice exhibit similarities with a break in the envelope gene of Cas-Br-E (Figure- 9). These analyses also suggest that amphotropic genomes are stable within the wild mouse population as was observed by our earlier *in vivo *experiments [4,30]. However, *experimental *inoculation of amphotropic retroviruses in the laboratory mice clearly results in genetic recombination with other MuLV sequences present in the chromosomal DNA of the inbred mice [4,28-30].

## Conclusion

The study presented herein represents the first comprehensive genomic analysis of an exogenously acquired, naturally occurring amphotropic retrovirus MuLV-1313 and it demonstrates that the molecular, biological and phylogenetic properties of this virus are distinct from a broad range of ecotropic, xenotropic or polytropic MuLV strains isolated from the inbred laboratory or other feral mice.

Analyses and comparison of each nucleotide of the full-length MuLV-1313 genome with those of the corresponding nucleotide of other MuLV strains indicate that this retrovirus is most closely related in the *gag *and *pol *genes (not the *env *gene) of another naturally occurring but a biologically distinct ecotropic retrovirus (Cas-Br-E), isolated from the wild mouse of another Southern California locale. Phylogenetic analyses of each gene separately and collectively as the whole genome, provide additional evidence that the "highly conserved" *gag *and *pol *genes of the two distinct retroviruses (amphotropic and ecotropic) of the Southern California wild mice are genetically related to each other compared to all known MuLV strains.

Analyses of full length genomes by the use of various bioinformatics programs such as SimPlot, has been useful in distinguishing genetic recombination in any part of the HIV genome. These programs have been useful in identifying genetic events that may occur between the genomes of replication competent retroviruses, host cellular sequences or other sequences derived from endogenous or exogenously infecting viruses. These recombinant viruses can be highly pathogenic, less pathogenic or could be associated with distinct oncogenic, neurologic and other diseases [4,28-30]. A thorough analysis of the entire MuLV-1313 genome indicates that this naturally occurring lymphomagenic retrovirus is not a recombinant of any of the numerous MuLV strains isolated globally.

Although the amphotropic MuLV-1313 and ecotropic Cas-Br-E MuLV are positioned at different branches of the evolutionary tree, both viruses have a unique origin (i.e. the same ancestor) as they initiate from the same node. Since both the amphotropic and ecotropic retroviruses have been maintained as infectious agents among the indigenous population of the Southern California feral mice, they have evolved into different host range groups by an evolutionary drift from the large family of genetically transmitted (i.e. endogenous) and non-genetically transmitted (i. e. exogenously acquired) MuLV strains of both inbred and feral mice.

## Methods

### Source of Virus

The amphotropic MuLV-1313 was isolated from a lymphomatous tumor of a feral mouse (*Mus musculus domesticus*) and cultured directly in several cell lines [2] including the human rhabdomyosarcoma (RD) cells [76]. The virus-infected cells (designated RD1313) were grown in Dulbecco's modified Eagle's medium with high glucose (Gibco BRL, Grand Island, NY) supplemented with 10% heat-inactivated fetal bovine serum (FBS) (GIBCO-BRL), 20 mM glutamine (Sigma Chemical Co., St. Louis, MO) and 50 ug ml^-1 ^gentamicin sulfate (United States Biochemical Corp., Cleveland, OH). Cell cultures were maintained *in vitro *at 37°C in a humidified atmosphere of 5% CO_2 _and genomic DNA was extracted from approximately 2.0 × 10^7 ^RD1313 cells using a QIAamp Blood Kit (QIAGEN Inc., Chatsworth, CA) according to the manufacturer's recommendations.

### Cloning of proviral DNA

The proviral DNA of MuLV-1313 was amplified from the chromosomal DNA of RD1313 cells by utilizing the Expand Long Template PCR System (Boehringer Mannheim) according to the manufacturer's suggestions. Prior to amplification of the MuLV-1313 genome, highly conserved regions of the MuLV genomes were identified by alignment of several complete MuLV genome sequences (AKV – accession number J01998, Moloney – J02255, Friend – M93134 and Cas-Br-E – X57540) and two sets of PCR primers were identified for the amplification of two overlapping proviral DNA fragments of the MuLV-1313 genome from the RD1313 cellular DNA. Briefly, 500 ng of high molecular weight cellular DNA was used as a template in an amplification reaction consisting of 2.5 U Taq/Pwo DNA polymerase mixture, 350 uM of each dNTP and 300 nM of each primer, all in a final 50 ul volume. The cycling parameters consisted of a single incubation at 94°C for 2 min. followed by 10 cycles of 94°C for 10 sec., 60°C for 30 sec. and 68°C for 4 min.; 20 cycles of 94°C for 10 sec., 60°C for 30 sec., and 68°C for 4 min. with increases of 20 sec. per cycle; and a final 68°C extension for 10 min. The 5' end of the MuLV 1313 genome was amplified utilizing U3-F (5'-TGGGCCAAACAGGATATCTGTGG-3', corresponds to coordinates ^#^7945–7967 of the Moloney MuLV genome) as the upstream primer and Pol-R (5'-GCGTTAATTTAGTTAAAGTCTCCTTG-3', coordinates ^#^5313–5288) as the downstream primer. The 3' end of the MuLV 1313 genome was amplified using Pol-F (5'-TTGCCAGAAACAGGTCAAGTATCTGG-3', ^#^3380–3405) as the upstream primer and U5-R (5'-TGACGGGTAGTCAATTACTCCGAG-3', ^#^129–106) and the cycling conditions were the same as those used to amplify the 5' half except that the initial annealing temperature was 57°C instead of 60°C. All amplicons were cloned into pBluescript IISK(+) (Stratagene, La Jolla, CA) at the Hinc II site after the repair of DNA ends by T4 DNA polymerase and agarose gel purification of the fragments.

### DNA Sequence Analysis

To facilitate sequencing of the cloned MuLV-1313 DNA fragments, nested sets of unidirectional, deletion mutants were generated for both the 5'– and 3'– amplicons using an Erase-A-Base kit (Promega Corp.) according to the manufacturer's instructions. Plasmid DNA's from randomly selected clones were subjected to restriction enzyme analysis and a set of subclones was chosen that differed from each other by 400–500 bp spanning the entire lengths of the respective MuLV-1313 inserts. A SequiTherm Long-Read cycle sequencing kit-LC (EpiCenter Technologies Corp., Madison, WI) was used according to the manufacturer's instructions to perform all sequencing reactions and the resulting products were subjected to gel electrophoresis on 6% polyacrylamide gels (Long-Ranger). The kit utilized a combined thermal cycle labeling and sequencing procedure employing the dideoxy chain terminator method. All sequencing reactions used fluorescent dye, 1.5 pmol of T3 or T7 labeled primers and 300–350 fmol of cloned, double-stranded plasmid DNA. Cycle sequencing parameters consisted of a single incubation of 95°C for 3 min. followed by 30 cycles of 95°C for 30 sec., 57°C for 15 sec. and 70°C for 30 sec. All sequencing data was obtained using an automated, LI-COR DNA sequencer, model 4200 (LI-COR, Lincoln, NE) with Base Image IR (version 4.0) image analysis software. Raw sequencing data were assembled into the final contig using the AlignIR (version 1.0) software package (LI-COR).

The nucleotide sequence of the complete MuLV 1313 genome has been deposited with the GenBank database under the accession number (AF411814).

### Multiple sequence alignment and Phylogenetic analyses

Computer-assisted searches for DNA sequence similarities to the MuLV-1313 genome were performed using the BLAST (Basic Local Alignment Search Tool) version 2.0 [77][78] that is maintained on the National Center for Bioinformatics Institute (NCBI). Sequences were aligned using the algorithm of Needleman and Wunsch [63] as implemented in the Gap program of the Wisconsin Package (version 9.0), Genetics Computer Group (GCG), Madison, WI [79]. The Gap program creates a global alignment between the two sequences that maximizes the number of matched residues and minimizes the number and size of gaps.

Phylogenetic analyses were performed using PHYLIP packages [66,79]. PHYLIP version 3.6. 2005, was distributed by Dr. J. Felsenstein, Department of Genome Sciences, University of Washington, Seattle. Other programs used included SEQBOOT, PROTDIST, DNADIST, NEIGHBOR, CONSENSE, and DRAWGRAM. First we applied bootstrapping technique to the original data set using SEQBOOT, which produced 100 bootstrapped data sets. The distance matrices on these data sets were achieved using PROTDIST for amino acid sequences and DNADIST for nucleotide sequences. The distance matrices were joined using NEIGHBOR. The tree files from NEIGHBOR were then applied with CONSENSE and the consensus was drawn using DRAWGRAM.

### Comparative Analysis of Full-length MuLV genomes

At present 15 full length MuLV genome sequences are available and an alignment of genomic sequences was generated by the Clustal W program [64,80]. Rooted phylogenetic trees were constructed from prealigned nucleic acid and amino acid sequences using different programs including CLUSTALTREE [65]. Evolutionary trees were generated in two steps; first, a distance matrix was established by calculating distances (percent divergence) between all pairs of sequences in the multiple alignments and second the Neighbor Joining method was applied to the distance matrix 149;50. All gaps were stripped from the alignments before the phylogenetic trees were constructed and bootstrapping was set at 1000. Viruses and their complete genomes used in this analysis are: MuLV 1313 (AF411814), MCF 1233 (U13766), AKV (J01998), Cas-Br-E (X57540), Friend 57 (X02794), Friend (FB29) (Z11128), Friend (FrC6-A8F5) (D88386), Friend (PVC-211) (M93134), Moloney (J02255), Rauscher (U94692), SRS 19-6 (AF019230), HEMV (AY818896), RadLV (K03363), MDEV (AF053745) and SL3-3 (AF169256).

For **dot matrix analyses**, multiple sequence alignment were made using Vector NTI (Invitrogen, Carlsbad, California) with default gap opening penalty of 15 and default gap extension penalty of 6.66. COMPARE produced point files ranging in size from 3528 points (*env *genes only) to 15621 points of full-length MuLV genomes. Dot matrix was constructed using Vector NTI tool with windows setting at 21 and stringency at 44.

The **SimPlot program **[72], was used to plot similarity versus position and it calculates percent identity of a query sequence to a panel of reference sequences after their alignment in a sliding window, which is moved across the alignment in steps. This is an interactive 32-bit software in which the windows and step sizes are adjustable. SimPlot was available as shareware from the author, Stuart C. Ray, M.D., 1999, SimPlot for Windows (version 2.5), John Hopkins University School of Medicine, Baltimore, MD, that has been used to identify inter-subtype recombinants of HIV genomes [72].

## Authors' contributions

SR conceived and supported the study. TH carried out complete genomic sequence analyses and bulk of experimental work with technical help from MW and ZS; YW conducted additional bioinformatics and phylogenetic analyses and both TH and SR drafted the manuscript.

## Supplementary Material

Additional File 1 This Similarity Plot is exactly the same as Figure 9 except that it was constructed using all 15 full-length. MuLV genomes which included Friend (FrC6-A8F5 D88386), (HEMV (AY818896), MDEV. (AF053745) and RadLV (K03363) in addition to those listed in the legend of Fig. 9. Note that no. similarity is observed with these additional viruses as evident by broken lines. Click here for file

Additional File 2 **Dot Martrix analyses of the whole MuLV-1313 genome**. Dot plots of the MuLV-1313 genome (GenBank accession number AF411814) were constructed utilizing the COMPARE which produced files of 15621 points of full-length MuLV genomes. Dot matrix was constructed using DOTPLOT programs of the Wisconsin Package, Version 9.0, Genetics Computer Group (GCG), Madison WI and Vector NTI (Invitrogen, Carlsbad, California) tool with windows setting at 21 and stringency at 44. This analysis compares each nucleotide position with the corresponding position of another genome (Dot). Solid diagonal line represents similarity and broken lines indicate gaps. Although Dot-Matrix analyses were performed on several MuLV strains, viruses that showed high similarity scores are shown in **panels A, B and C. Dot Matrix **analyses of full-length genomic sequences shown include ; **Panel A, **Cas-Br-E [25] (X57540); **Panel B, **AKV (J01998), and **Panel C, **Moloney (J02255). The highest nucleotide similarity is observed with the CAS-Br-E ecotropic virus isolated from a Southern California Wild mouse with paralysis **(Panel A). **This is followed by Moloney [8] and AKV MuLV strains [85] **(Panels B & C respectively)**. Note, the *env *sequences of MuLV-1313 are totally unrelated to all three viruses shown by large gap in this area of the diagonal line. In addition, note the numerous broken lines in *gag *and *pol *regions of the Moloney and AKV MuLV genomes. Click here for file
