# Supplementary figures and images for: Molecular and phylogenetic analyses of a new Amphotropic murine leukemia virus (MuLV-1313)
Source: Virol J. 2006 Dec 5;3:101. doi: 10.1186/1743-422X-3-101 (PMC1769482; doi:10.1186/1743-422X-3-101)

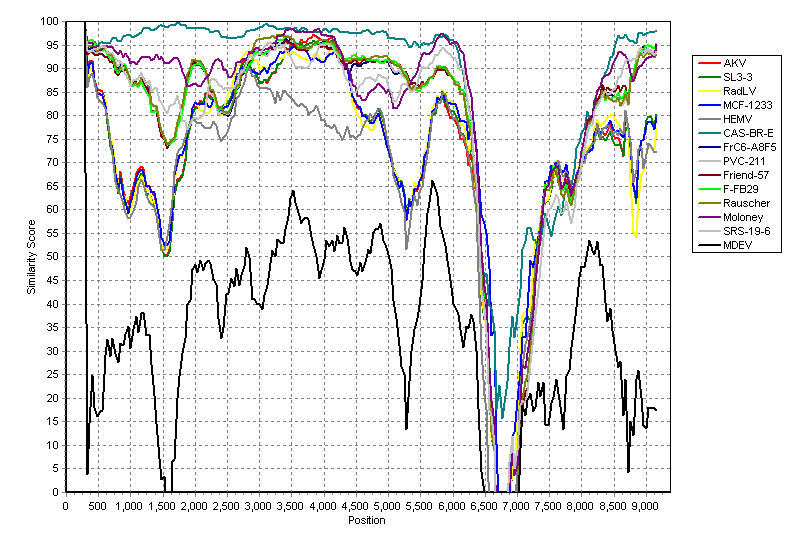

Supplement: Additional File 1 — This Similarity Plot is exactly the same as Figure 9 except that it was constructed using all 15 full-length. MuLV genomes which included Friend (FrC6-A8F5 D88386), (HEMV (AY818896), MDEV. (AF053745) and RadLV (K03363) in addition to those listed in the legend of Fig. 9. Note that no. similarity is observed with these additional viruses as evident by broken lines. [file 1743-422X-3-101-S1.doc]

**
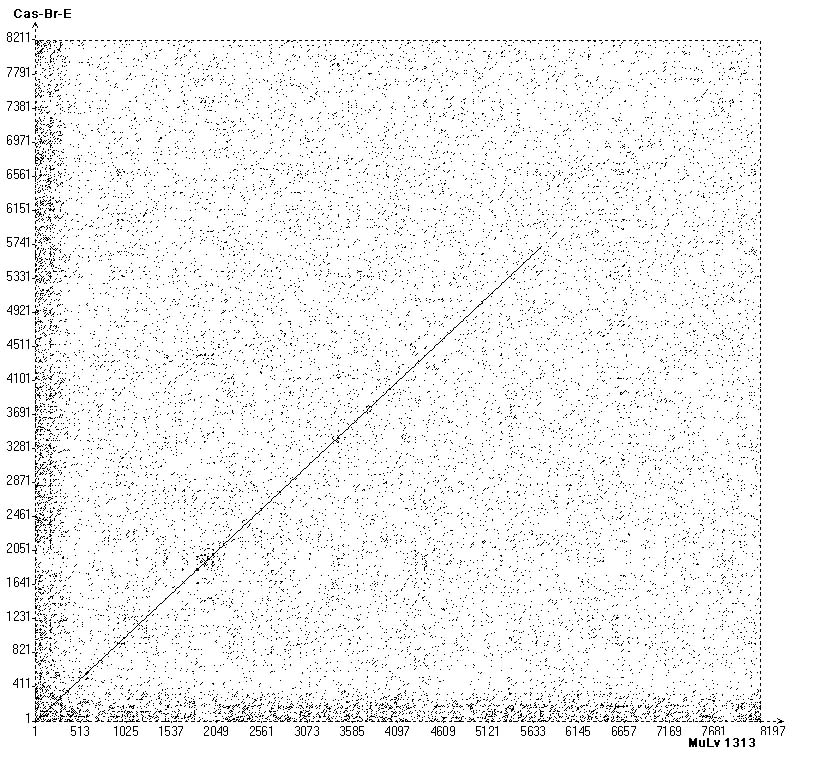

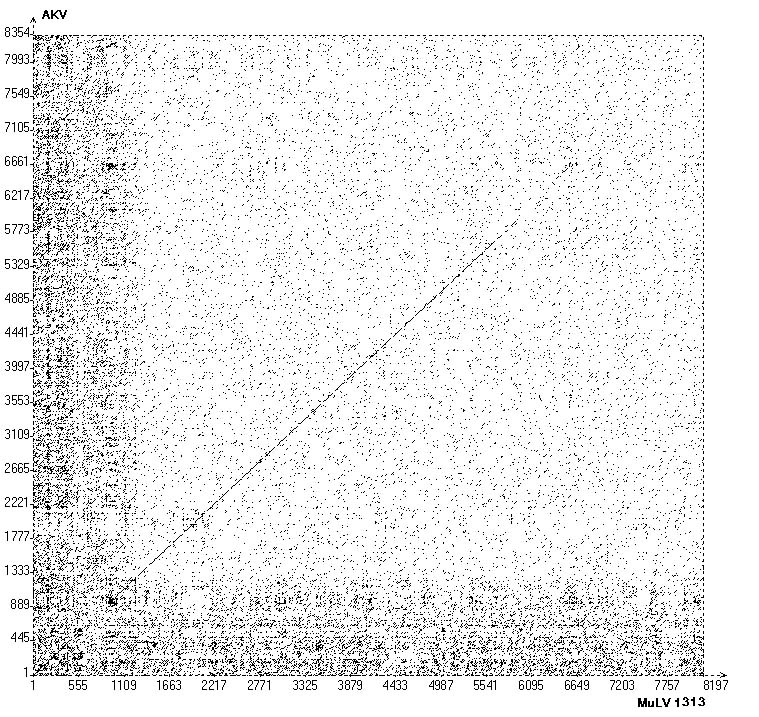

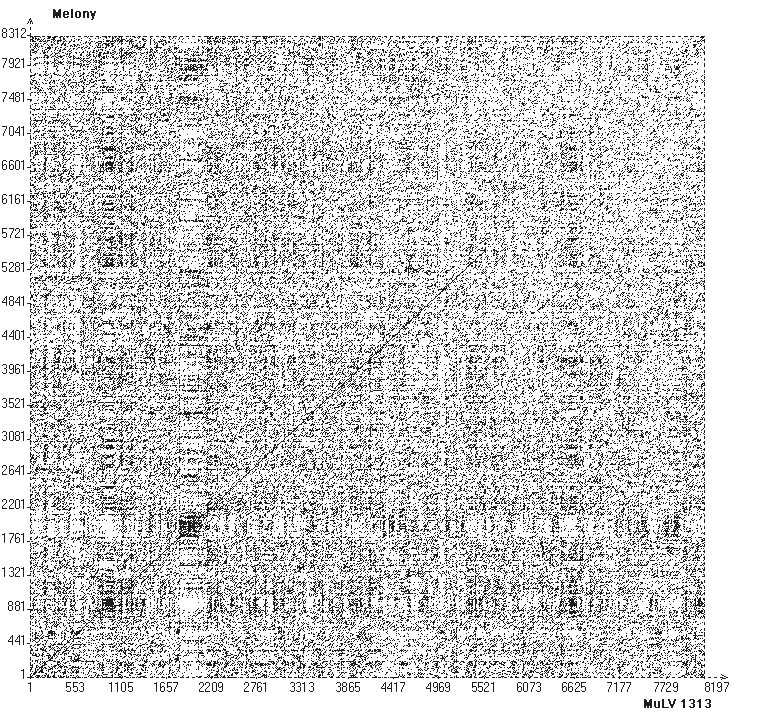
**

Supplement: Additional File 2 — Dot Martrix analyses of the whole MuLV-1313 genome. Dot plots of the MuLV-1313 genome (GenBank accession number AF411814) were constructed utilizing the COMPARE which produced files of 15621 points of full-length MuLV genomes. Dot matrix was constructed using DOTPLOT programs of the Wisconsin Package, Version 9.0, Genetics Computer Group (GCG), Madison WI and Vector NTI (Invitrogen, Carlsbad, California) tool with windows setting at 21 and stringency at 44. This analysis compares each nucleotide position with the corresponding position of another genome (Dot). Solid diagonal line represents similarity and broken lines indicate gaps. Although Dot-Matrix analyses were performed on several MuLV strains, viruses that showed high similarity scores are shown in panels A, B and C. Dot Matrix analyses of full-length genomic sequences shown include ; Panel A, Cas-Br-E [25] (X57540); Panel B, AKV (J01998), and Panel C, Moloney (J02255). The highest nucleotide similarity is observed with the CAS-Br-E ecotropic virus isolated from a Southern California Wild mouse with paralysis (Panel A). This is followed by Moloney [8] and AKV MuLV strains [85] (Panels B & C respectively). Note, the env sequences of MuLV-1313 are totally unrelated to all three viruses shown by large gap in this area of the diagonal line. In addition, note the numerous broken lines in gag and pol regions of the Moloney and AKV MuLV genomes. [file 1743-422X-3-101-S2.doc]
